# The RNA-binding protein quaking maintains endothelial barrier function and affects VE-cadherin and β-catenin protein expression

**DOI:** 10.1038/srep21643

**Published:** 2016-02-24

**Authors:** Ruben G. de Bruin, Eric P. van der Veer, Jurriën Prins, Dae Hyun Lee, Martijn J. C. Dane, Huayu Zhang, Marko K. Roeten, Roel Bijkerk, Hetty C. de Boer, Ton J. Rabelink, Anton Jan van Zonneveld, Janine M. van Gils

**Affiliations:** 1 Einthoven Laboratory of Experimental Vascular Medicine, Division of Nephrology, Department of Internal Medicine, Leiden University Medical Center, Leiden, the Netherlands

## Abstract

Proper regulation of endothelial cell-cell contacts is essential for physiological functioning of the endothelium. Interendothelial junctions are actively involved in the control of vascular leakage, leukocyte diapedesis, and the initiation and progression of angiogenesis. We found that the RNA-binding protein quaking is highly expressed by endothelial cells, and that its expression was augmented by prolonged culture under laminar flow and the transcription factor KLF2 binding to the promoter. Moreover, we demonstrated that quaking directly binds to the mRNA of VE-cadherin and β-catenin and can induce mRNA translation mediated by the 3′UTR of these genes. Reduced quaking levels attenuated VE-cadherin and β-catenin expression and endothelial barrier function *in vitro* and resulted in increased bradykinin-induced vascular leakage *in vivo*. Taken together, we report that quaking is essential in maintaining endothelial barrier function. Our results provide novel insight into the importance of post-transcriptional regulation in controlling vascular integrity.

All blood vessels are lined with a single layer of endothelial cells (ECs), which form a vital barrier between the blood and underlying tissue. The control of the EC barrier is critical to maintain vascular stability and retain circulating fluids, solutes, proteins and cells within the vasculature. Excessive vascular permeability plays a key role in many pathophysiological conditions such as septic shock, vascular leakage, hypertension, edema and atherosclerosis^1^,^2^. Inter-endothelial contacts within the monolayer must therefore be maintained for proper barrier function, while on the other hand, the cellular junctions must be sufficiently plastic to allow the growth and development of blood vessels, as well as the passage of leukocytes to the underlying tissue in case of an inflammatory reaction^3^.These specialized endothelial cell-cell adhesions are mediated by adherens, tight- and gap junctions^2^. In ECs, cell-cell interactions at adherens junctions are facilitated by the transmembrane protein VE-cadherin, of which the extracellular domain forms a zipper-like structure between the cells^4^. The intracellular domain of VE-cadherin is linked to the actin cytoskeleton via a complex array of structural and signaling proteins, including β-, γ-, α- and p120-catenins^2^.

In recent years, the importance of post-transcriptional control of gene expression in EC biology has become increasingly evident. For instance, microRNAs, such as the EC-enriched microRNA-126, were shown to play a critical role in vascular integrity and EC homeostasis^5^,^6^,^7^. Next to microRNAs, non-coding RNAs such tie-1AS, MIAT and TUG1, are also novel players in endothelial function^8^,^9^,^10^,^11^. So far, RNA-binding proteins (RBP) have attracted less attention as essential post-transcriptional regulators of RNA fate. The human genome encodes an estimated 424 RBPs many of which function in post-transcriptional regulation driving alternative splicing of pre-mRNAs, nuclear export or retention, RNA stability and degradation, sequestration in granules and, ultimately, the translation into protein^11^. RBPs can mediate RNA and protein expression by binding to the 3′UTR of target mRNAs^12^. RBPs are classified by their capacity to directly interact with RNA through RNA-binding domains such as the RNA-Recognition-Motif or the K-Homology Domain. Similar to transcription factors binding to DNA motifs, RBPs can recognize and bind to their targets by recognizing specific RNA sequence motifs^13^.

Given the large repertoire of RBPs present in any given cells, little is known about the role of RBPs in endothelial cell biology. The double-stranded RBP 76/NF70 was shown to facilitate vascular endothelial growth factor expression under hypoxic conditions by promoting vascular endothelial growth factor mRNA loading onto polysomes^14^. Also, splicing factor 2 regulates alternative splicing of the endothelial endoglin gene^15^ and stabilization of the Sirtuin 1 mRNA by the RBP HuR was shown to repress the endothelial inflammatory response^16^. The RNA-binding protein Quaking (QKI) may also be highly relevant for endothelial function as it was shown to be essential for embryonic blood vessel development and visceral endoderm function^17^,^18^, and has also been implicated in angiogenesis^19^. While, QKI was originally identified for its role in myelination of the central and peripheral nervous system, later studies revealed that the RBP QKI is more ubiquitously expressed^20^. There are three major QKI protein isoforms; QKI-5, QKI-6, and QKI-7 that require to either homo- or hetero-dimerize in order to bind RNA^21^. The post-transcriptional events published to be orchestrated by QKI include pre‐mRNA splicing, mRNA export, mRNA stability and translation^22^,^23^,^24^,^25^,^26^. Consequently, QKI is involved in multiple cellular processes such as cellular differentiation, apoptosis, proliferation and migration^20^. Upon vascular injury, we have previously demonstrated that QKI plays a critical role in the proliferation and contractility of vascular smooth muscle cells by mediating the alternative splicing of the transcription factor myocardin^27^. During these studies, we noted that QKI is highly enriched in the endothelium of healthy control arteries. Therefore we assessed whether QKI contributes to endothelial function *in vitro* and *in vivo*. Here we identify QKI as a novel post-transcriptional regulator of expression of both VE-cadherin and β-catenin and as an essential regulator of EC barrier function.

## Results

### Quaking is expressed in macro- and micro-vascular endothelial cells

**Figure 1:**
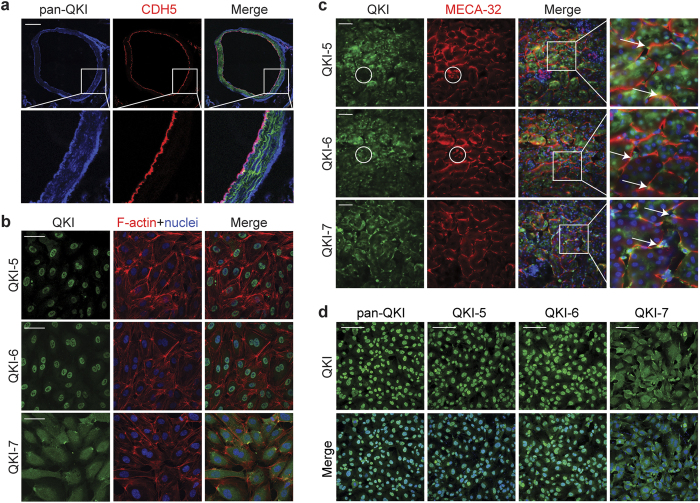
Quaking is highly expressed in macro- and micro-vascular endothelial cells. (**a**) Immunofluorescent staining of pan-QKI (blue) and endothelium marker (red, VE-cadherin-CreER/Rosa^tdTomato^) in mouse aorta sections. Scale bar 200 μm. (**b**) Immunofluorescent staining of QKI-5, QKI-6 or QKI-7 (green), F-actin (red) and DAPI (blue) in HUVECs. Scale bars 50 μm. (**c**) Immunofluorescent staining of QKI-5, QKI-6 or QKI-7 (green), endothelium marker (red, MECA-32) and DAPI (blue) in mouse kidney sections. Arrows indicate peritubular capillaries, circles indicate glomeruli. Scale bars 50 μm. (**d**) Immunofluorescent staining of pan-QKI, QKI-5, QKI-6 or QKI-7 (green), and DAPI (blue) in MVECs. Scale bars 100 μm.

Given our previous observation that QKI is expressed in the endothelium of healthy human arteries^27^, we first assessed QKI expression in ECs of the native macrovascular vessels *in vivo*. For this we performed immunostaining of QKI on aortic sections of VE-cadherin-CreER/Rosa^tdTomato^ reporter mice. QKI protein was highly enriched in ECs as compared to medial smooth muscle cells in the healthy artery as evidenced by its co-localization with the endothelial specific marker VE-cadherin (Fig. 1a). In the deeper layers of the adventitia, perivascular fibroblasts were also found to stain positive for QKI. To confirm whether QKI was also expressed in cultured human macrovascular ECs we assessed mRNA and protein levels of the three major isoforms of QKI in cultured human umbilical vein endothelial cells (HUVECs). The mRNAs of all three isoforms, QKI-5, QKI-6 and QKI-7 were abundantly expressed with the most abundant isoform Qki5 expressed at a similar level as the endothelial Nos3 gene under static culture conditions (Supplementary Fig. 1a). Also, western blot readily confirmed the presence of the corresponding QKI protein isoforms in the lysates of the cells (Supplementary Fig. 1b). As earlier reports showed distinct cellular localization of the QKI protein isoforms in glial cells^28^ and Hela cells^29^ we performed immunostaining for the QKI isoforms in the cultured ECs. Indeed, confirming these previous reports, QKI-5 and QKI-6 expression was highly enriched in the nuclei while QKI-7 displayed a cytoplasmic localization (Fig. 1b).

We next assessed QKI expression in ECs of the microvasculature by immunostaining of the QKI protein isoforms in mouse kidney sections and cultured human pulmonary microvascular endothelial cells (MVECs). Co-staining for the mouse endothelial antigen-23 (MECA32) clearly demonstrated QKI expression in the microvascular beds including the peritubular capillaries (Fig. 1c, arrows) and to a lesser extent the glomerular endothelium (Fig. 1c, circles). Evident staining was also observed in the kidney epithelium. Moreover, similar to HUVECs, distinct sub-cellular localization of the QKI-protein isoforms was observed in MVECs (Fig. 1d). Interestingly, particularly at higher magnification, in both HUVECs and MVECs, QKI-7 appeared to be focally enriched at the plasma membrane. Staining with an antibody that recognizes all three protein isoforms (pan-QKI) showed that the vast majority of QKI protein is localized in the nucleus (Fig. 1d). Taken together, these results demonstrate that QKI is highly expressed in the endothelium of both the macrovascular as well as in the microvascular beds.

### Laminar shear stress induces QKI expression

**Figure 2:**
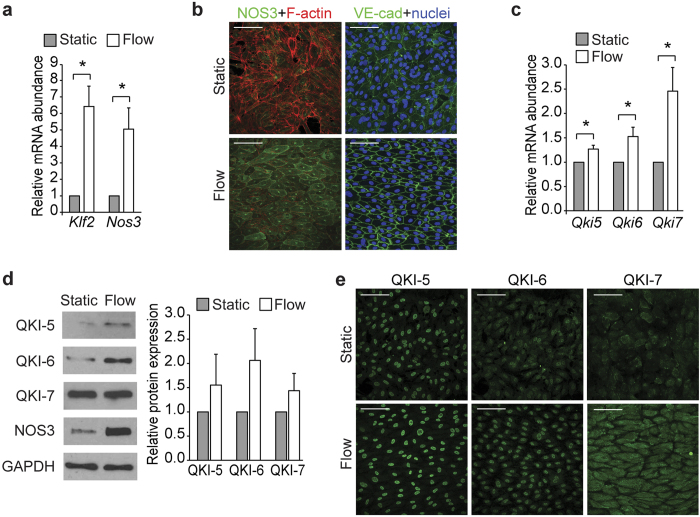
Endothelial cells cultured under laminar flow show increased QKI expression. (**a**) Quantitative RT-PCR analysis of Klf2 and Nos3 mRNA isolated from HUVECs cultured under static or laminar flow conditions. Results are presented relative to static cultured cells, set as 1. Mean ± s.e.m of *n* = 3–6. ^*^P < 0.05. (**b**) Immunofluorescent staining of NOS3 (green, left panels) or VE-cadherin (green, right panels) and nuclei (blue) or F-actin (red) in HUVECs cultured under static or laminar flow conditions. Scale bars 100 μm. (**c**) Quantitative RT-PCR analysis of Qki5, Qki6 and Qki7 mRNA isolated from HUVECs cultured under static or laminar flow conditions. Results are presented relative to static cultured cells, set as 1. Mean ± s.e.m of *n* = 6–7. ^*^P < 0.05. (**d**) Immunoblot analysis of QKI-5, QKI-6, QKI-7, NOS3 or GAPDH (loading control) in protein lysates of HUVECs cultured under static or laminar flow conditions. Bar graph shows quantification of 3 independent experiments, mean ± s.e.m. Results are relative to static cultured cells, set as 1. (**e**) Immunofluorescent staining of QKI-5, QKI-6 or QKI-7 (green) in HUVECs cultured under static or laminar flow conditions. Scale bars 100 μm.

Depending on their location in the vascular tree, ECs are exposed to varying magnitudes and types of shear stress caused by the flowing blood. It has been well established that the type of shear stress, either by laminar or disturbed flow, drives distinct cellular signaling pathways resulting in either quiescent or inflammatory EC phenotype, respectively^30^. Given the marked expression of QKI in ECs in healthy vessels, we postulated that vascular protective hemodynamic conditions may drive the expression of QKI. To test this, we cultured ECs under laminar shear for 7 days (10 dyne/cm^2^). Compared to statically cultured control cells, ECs exposed to laminar flow showed higher expression of the shear-responsive genes Nos3 and Krüppel-Like Factor 2 (Klf2)^31^,^32^ (Fig. 2a,b). Consistent with this quiescent EC phenotype, laminar flow exposed ECs showed well organized VE-cadherin at the cell-cell junctions, as well as short dense shear-fiber formation and alignment to the direction of the flow (Fig. 2b). Using this culture system, we determined whether prolonged laminar flow could also induce QKI expression. Quantitative RT-PCR revealed that the mRNA of all three Qki isoforms is indeed augmented by a shear dependent mechanism (Fig. 2c). Moreover, immunoblot analysis and fluorescent immunostaining of control (static) and 7 days sheared ECs revealed an evident increase in QKI protein expression (Fig. 2d,e). Interestingly, the mRNA and protein levels of the cytoplasmic isoform QKI-7 showed the most prominent increase upon prolonged laminar flow.

**Figure 3:**
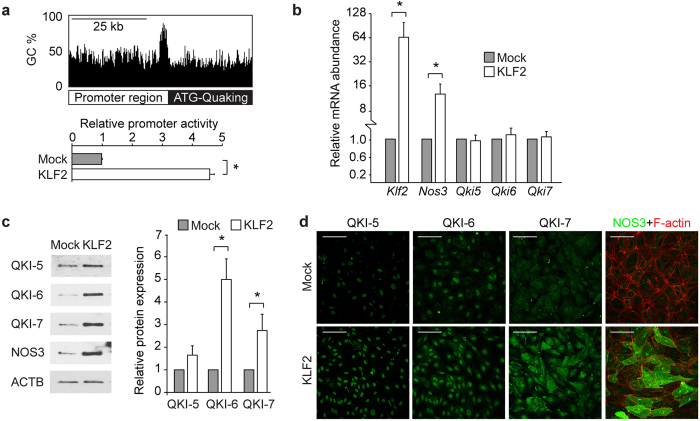
Quaking is induced by KLF2. (**a**) Schematic representation of the promoter of QKI gene, demonstrating a high GC content (upper panel). Bar graph shows QKI promoter-luciferase reporter activity in HEK293T cells after transduction with lenti-KLF2 virus or lenti-mock virus. Results are presented relative to reporter activity in mock transduced cells, set as 1. Mean ± s.d. from one experiment representative of three independent experiments. ^*^P < 0.05. (**b**) Quantitative RT-PCR analysis of Klf2, Nos3, Qki5, Qki6 and Qki7 mRNA isolated from HUVECs transduced with mock or KLF2 overexpressing lenti-virus. Results are presented relative to cells transduced with mock virus, set as 1. Mean ± s.e.m. of *n* = 4. ^*^P < 0.05. (**c**) Immunoblot analysis of QKI-5, QKI-6, QKI-7, NOS3 or ACTB (loading control) in protein lysates of HUVECs transduced with mock or KLF2 overexpressing lenti-virus. Bar graph shows quantification of 3 independent experiments, mean ± s.e.m. Results are relative to static cultured cells, set as 1. *P < 0.05. (**d**) Immunofluorescent staining of QKI-5, QKI-6, QKI-7 or NOS3 (green) and F-actin (red, rights panels) in HUVECs cultured transduced with mock or KLF2 overexpressing lenti-virus. Scale bars 100 μm.

To assess the molecular pathway by which QKI expression is augmented under laminar flow conditions we examined the promotor region of QKI. This revealed a prominent GC-rich DNA stretch, encompassing 78.1% GC content in the 1000 base pairs upstream of the QKI ATG start site with many CpG sites present (Fig. 3a, upper illustration). The shear induced transcription factor KLF2 binds to GC-rich DNA sequences^33^ and has been described to drive a laminar shear dependent anti-inflammatory gene expression profile in EC^34^,^35^. We utilized a QKI promoter-luciferase reporter gene to test whether QKI expression could be directly induced by binding of KLF2 to the promoter region. Co-transfection of this reporter gene with the KLF2 cDNA in HEK293T cells resulted in an induction of the activity of the QKI promoter region (Fig. 3a, bar graph). Next, we tested whether lentiviral overexpression of KLF2 in HUVECs could also induce QKI expression. Overexpression of KLF2 resulted in increased expression of Nos3 mRNA, but did not result in altered Qki mRNA levels (Fig. 3b). In contrast, overexpression of KLF2 did result in a marked increase in QKI protein levels, as evidenced by immunostaining and immunoblot analysis (Fig. 3c,d). NOS3 protein detection was taken along for validation that a functional KLF2 was overexpressed (Fig. 3b–d). These experiments support the hypothesis that QKI protein expression can be mediated by the transcription factor KLF2, either through direct interaction with the QKI promoter region and/or through additional post-transcriptional mechanisms. Taken together, we show that QKI expression is particularly expressed in quiescent, healthy ECs.

### Quaking binds the VE-cadherin and β-catenin mRNA and promotes translation

**Figure 4:**
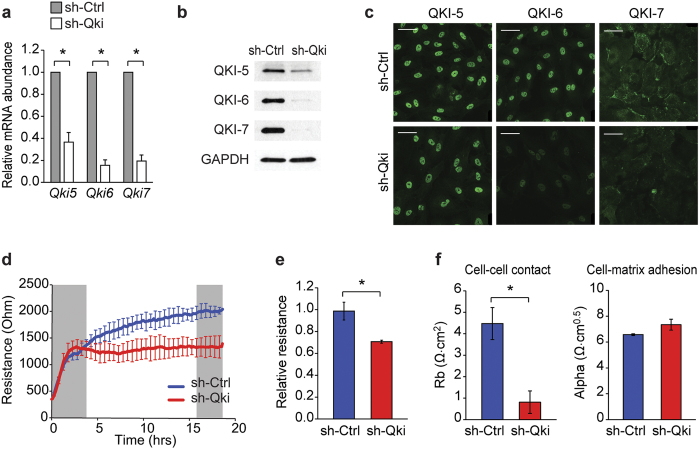
Targeting QKI perturbs endothelial barrier function *in vitro*. (**a**) Quantitative RT-PCR analysis of Qki5, Qki6 and Qki7 mRNA isolated from HUVECs transduced with anti-Qki shRNA (shQKI) or control shRNA (shCtrl). Results are presented relative to shCtrl, set as 1. Mean ± s.e.m. of *n* = 5. ^*^P < 0.05. (**b**) Immunoblot analysis of QKI-5, QKI-6, QKI-7 or GAPDH (loading control) in protein lysates of shCtrl or shQKI HUVECs. (**c**) Immunofluorescent staining of QKI-5, QKI-6 or QKI-7 (green) in shCtrl or shQKI HUVECs. Scale bars 50 μm. (**d**) Transendothelial electrical resistance of shCtrl or shQKI transduced HUVECs seeded on ECIS electrodes. Left grey box indicates time frame of adhesion and spreading, right grey box indicates time frame of stable monolayer. (**e**) Relative transendothelial electrical resistance of shCtrl or shQKI HUVEC stable monolayer, time frame indicated by right grey box in d, shCtrl set so 1. Mean ± s.e.m. of *n* = 3. ^*^P < 0.05. (**f**) Absolute endothelial electric resistance attributable to cell-cell contact (Rb, left bar graph) and cell-matrix interaction (Alpha, right bar graph) of shCtrl or shQki HUVECs. Mean ± s.d. of *n* = 2. ^*^P < 0.05.

To gain insight into the function of QKI in ECs we silenced QKI expression in ECs using lentiviral shRNA vectors targeting the QKI mRNA (shQKI) and a non-QKI targeting control shRNA (shCtrl). Repression of QKI expression was validated using qRT-PCR, immunoblot analysis and immunofluorescent staining (Fig. 4a–c). Next, shCtrl- and shQKI-treated ECs were seeded on gelatin-coated culture chambers that have electrodes present in the growth area to facilitate the measuring of electrical resistance by Electric Cell-substrate Impedance Sensing (ECIS)^36^. Targeted reduction of QKI in ECs did not affect the capacity of the cells to adhere and spread (Fig. 4d), but did result in the inability to form a proper high resistance monolayer as compared to control cells (Fig. 4d,e). Applying further mathematical modeling using the provided ECIS software, this modeling uses the impedance data to calculate the cell morphological parameters cell-cell contact and cell-matrix interaction^37^,^38^, it was confirmed that QKI reduction potently and specifically attenuated the endothelial cell-cell interactions (Fig. 4f).

**Figure 5:**
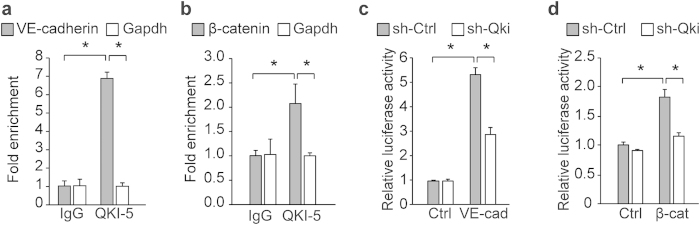
Quaking protein binds to VE-cadherin and β-catenin mRNA and affects translation. (**a**,**b**) RNA-immunoprecipitation in HUVECs using an IgG control or QKI-5 antibody. VE-cadherin (**a**), β-catenin (**b**) or Gapdh mRNA abundance in immune-precipitated fraction was determined by qRT-PCR. Results are presented relative to IgG immunoprecipitation, set as 1. Mean ± s.d. from one experiment representative of three independent experiments. ^*^P < 0.05. (**c**,**d**) VE-cadherin (**c**), β-catenin (**d**) or Control (Ctrl) 3′UTR luciferase-reporter constructs were co-transfected with shCtrl or shQKI in HEK293T cells and luciferase activity levels measured. Results are presented relative to Ctrl 3′UTR in shCtrl cells, set as 1. Mean ± s.d. from one experiment representative of three independent experiments. ^*^P < 0.05.

Given the RNA-binding properties of QKI, we postulated that the effects on barrier function could be explained by the post-transcriptional regulation of the mRNAs of genes involved in cell-cell interactions. To identify potential binding partners of QKI that are involved in barrier function, we examined the previously published gene list of 1430 potential target genes that have been computationally determined to contain a Quaking Response Element (QRE) (NACUAAY-N1–20-UAAY)^39^. Interestingly, VE-cadherin and β-catenin, essential for endothelial adherent junctions, both were shown to harbor high-affinity QREs in the 3′UTRs of their mRNA (Supplementary Fig. 2). Next to this we as well examined the published QKI PAR-CLIP in HEK293 cells^40^, as well as the pre-mRNA sequence of VE-cadherin, since VE-cadherin is not expressed in HEK293T cells (Supplementary Fig. 2). In intronic regions several QREs are present, however no alternative splicing events of the flanking exons have been annotated (UCSC or Refseq). Using RNA immuno-precipitation, we first assessed whether QKI could bind to the mRNA of VE-cadherin and β-catenin. Indeed qRT-PCR analysis confirmed that mRNAs of both VE-cadherin and β-catenin were highly enriched in the QKI-antibody precipitated RNA fraction both in HUVECs (Fig. 5a,b) as well as in MVECs (Supplementary Fig. 3a,b). Since the QREs in the VE-cadherin and β-catenin mRNAs were localized in their 3′UTRs, we next sought to determine whether, next to binding, QKI as well has an effect on protein translation. For this we utilized luciferase-reporter genes fused to the 3′UTRs of both genes. Indeed, the presence of a QRE in the 3′UTRs from both genes showed a clear increase in luciferase activity, while these effects were blunted when QKI was repressed (Fig. 5c,d). These data indicate that QKI does not only bind to the mRNA of VE-cadherin and β-catenin, but also increases the translation by binding to the 3′UTRs of these mRNAs.

### Reducing QKI predisposes to vascular leakage

**Figure 6:**
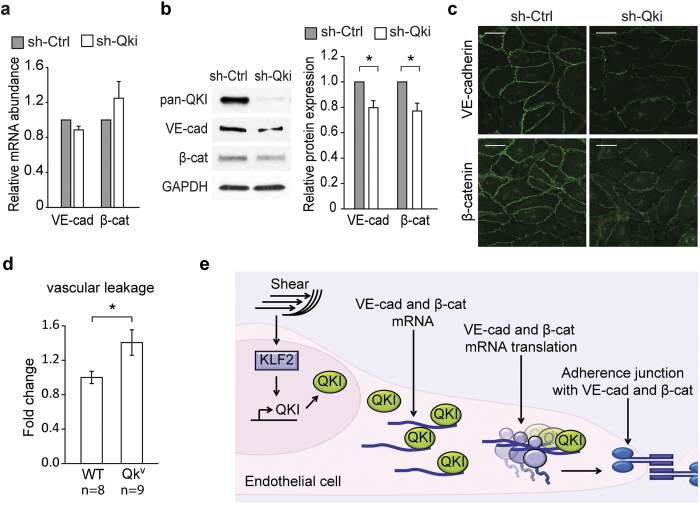
Abridged QKI results in reduced VE-cadherin and β-catenin and enhanced vascular leakage. (**a**) Quantitative RT-PCR analysis of VE-cadherin and β-catenin mRNA isolated from shCtrl or shQKI HUVECs. Results are presented relative to shCtrl, set as 1. Mean ± s.e.m. of *n* = 5. ^*^P < 0.05. (**b**) Immunoblot analysis of pan-QKI, VE-cadherin, β-catenin or GAPDH (loading control) in protein lysates of shCtrl or shQKI HUVECs. Bar graph shows quantification of 3 independent experiments, mean ± s.e.m. Results are relative to static cultured cells, set as 1. *P < 0.05. (**c**) Immunofluorescent staining of VE-cadherin or β-catenin in shCtrl or shQKI HUVECs. Scale bars 50 μm. (**d**) Vascular leakage upon Bradykinin (8 mg/kg) stimulation was measured in the gut microvasculature of WT and Qk^v^ mice, by spectrophotometric quantification of extravasated albumin that was labeled using Evans Blue. Results are presented relative to those of WT mice, set as 1. Mean ± s.e.m. of *n* = 8–9. ^*^P < 0.05. (**e**) A schematic diagram of endothelial QKI, induced by laminar flow and KLF2, in maintaining endothelial adherence junctions by binding to and inducing translation of VE-cadherin and β-catenin mRNA.

We next used ECs to validate that a reduction of QKI indeed decreases the abundance of VE-cadherin and β-catenin expression. While shRNA-mediated QKI knockdown in the EC did not reveal a pronounced reduction of either VE-cadherin or β-catenin mRNA (Fig. 6a), immunoblot analysis did show reduced VE-cadherin and β-catenin protein levels in ECs (Fig. 6b). In addition, immunofluorescence staining of VE-cadherin and β-catenin in shCtrl or shQKI ECs revealed that the expression of both VE-cadherin and β-catenin were diminished at the adherent junctions of shQKI treated cells (Fig. 6c). These data confirm that in ECs, QKI does not regulate mRNA transcript abundance, but instead mediates translation and thereby protein expression of VE-cadherin and β-catenin.

Finally, we postulated that decreased QKI expression would also result in impaired EC barrier function *in vivo*. To that end, we used the Quaking^viable^ (Qk^v^) mouse strain that has been widely used as a model to study the effects of decreased QKI levels. Vascular leakage and EC barrier function was assessed *in vivo* by Evans blue extravasation upon stimulation with bradykinin. Bradykinin is a well described potent permeability-increasing inflammatory mediator by signaling through the bradykinin receptors (BDKRB1 and BDKRB2)^41^ and Orsenigo *et al*. have shown VE-cadherin mediated vascular leakage in response to bradykinin^42^. Moreover, it has been reported that the mouse intestine microvasculature shows a strong susceptibility to bradykinin^43^. Therefore, we used a similar approach to robustly quantify extravasated Evans blue 5 minutes after intravenous injection of bradykinin. In concordance with our *in vitro* studies, a reduction of QKI expression *in vivo* indeed resulted in a significant 40% increase of Evans blue leakage upon bradykinin stimulation (Fig. 6d). These results further confirm that QKI expression is required for maintaining the integrity of EC barrier function *in vivo*.

## Discussion

In this study we provide evidence that the RBP QKI is highly expressed in quiescent ECs and is required for the maintenance of endothelial barrier function by increasing the expression of VE-cadherin and β-catenin in the intra-cellular junctions of the endothelium. Following the notion that the 3′UTRs of both the β-catenin and VE-cadherin mRNA contained QKI-binding sites, we investigated the role of QKI in the expression of these genes and demonstrated that the interaction of QKI with the 3′UTR region of VE-cadherin and β-catenin mRNA resulted in increased translation of these genes (Fig. 6e).

Previous reports demonstrated that the binding of QKI to the 3′UTR region of target mRNAs can affects mRNA stability and translation in a transcript and cell-specific fashion. For instance, in oligodendrocytes, QKI binding to the mRNAs of myelin basic protein (MBP)^44^ and its regulator Hnrnpa1^45^ was demonstrated to stabilize these mRNA levels by counteracting their rate of degradation. In contrast, QKI binding to the UTR of the Forkhead Box O1 mRNAs in cancer cells results in lower levels of this mRNA^46^,^47^. In contrast, our data in this study have indicated that in endothelial cells, binding of QKI to VE-cadherin and β-catenin 3′UTRs does not impact the mRNA levels, yet does enhance protein expression of VE-cadherin and β-catenin. Two previous reports have also implicated post-transcriptional regulation of β-catenin by QKI. In those studies adenovirus-mediated overexpression of QKI resulted in a decrease of protein expression, similarly without a change in mRNA abundance^47^,^48^. Together these results point to complex regulatory mechanisms, for example the direct competition of QKI and other RBPs binding to the 3′UTR, such as the RBPs Tristeraprolin^49^ or HuR^50^,^51^.

Increased expression of VE-cadherin and β-catenin following the binding of QKI to their 3′UTRs could also be explained by a direct competition for binding with microRNAs that would cause translational arrest without mRNA degradation. However, in silico analyses revealed no known microRNA binding sites at the QRE site in the 3′UTRs of VE-cadherin and β-catenin. Alternatively, increased expression of the QKI-bound mRNAs could be related to affect QKI-dependent localization and shuttling of these mRNAs. The homo- or hetero-dimerization dependent binding of RNA^52^,^53^, the distinct subcellular localization of the QKI isoforms^29^ and the presence of QKI in specific RNA containing granules^26^ are consistent with a role for QKI in the intracellular distribution of mRNA between the nucleus and various cytoplasmic compartments. For instance, nuclear retention was demonstrated for the MBP-1 mRNA in Qk^v^ mice^54^. Given the above, it is tempting to speculate that, in EC, QKI-7 containing heterodimers serve to shuttle bound mRNA to the cellular periphery and facilitate the local translation at for example adherens junctions.

Endothelial barrier function and the prevention of vascular leakage is tightly coupled to signaling pathways that are induced by laminar shear stress^55^. Our observation that ECs cultured for prolonged time under laminar flow, induced QKI mRNA and protein expression and most likely, influenced its junction-stabilizing functions, is consistent with this concept. Notably, lentiviral overexpression of the flow induced transcription factor KLF2^31^ did reveal an increased QKI protein expression as assessed by immunostaining and immunoblot. Therefore, our promoter activity experiments indicate that KLF2 mediates the promoter activity of QKI. This hypothesis was supported by the work of Redmond and co-workers who reported that, compared to control cells, QKI was one of the most repressed genes in the embryonic yolk sack erythroid cells of KLF2 knockout mice^56^. Together this points towards a regulation of QKI by KLF2, however it cannot be excluded that other parallel pathways are involved. For instance, several microRNA-mediated feedback loops have been described in literature, such as KLF2 driven microRNA-143 and microRNA-148 expression^57^ which, based on in silico analyses, are also predicted to bind to the 3′UTRs of the QKI mRNAs. Based on *in silico* analysis these microRNAs could target the QKI 3′UTRs, but not VE-cadherin and β-catenin, and induce mRNA degradation in HUVECs. Strikingly, genome wide *in silico* analyses suggested that the QKI 3′UTRs are probably extremely susceptible to microRNA regulation, ranking number 11 genome wide in the number of potential microRNA seed sequences present in its 3′UTR^58^. Combined with the induced QKI promoter activity by KLF2, microRNAs could result in an induction of QKI protein expression without an evident increase in QKI mRNA levels, as observed upon lentiviral KLF2 overexpression in HUVEC cells. The difference seen in QKI mRNA levels upon laminar flow and induced KLF2 expression, could be explained by other changes. Laminar flow induces not only KLF2, but also other signalling pathways that might contribute to the regulation of QKI mRNA and protein expression. Next to the change in microRNAs and QKI by KLF2 and/or laminar flow we do not exclude that the expression of other RNA-binding proteins might also be changed and capable of influencing QKI mRNA and protein levels.

Taken together, while previous studies demonstrated a critical role for QKI in embryonic blood vessel development by mediating intra-cellular interactions between ECs and the cells of the visceral endoderm, we now show that the RNA-binding protein QKI also serves a postnatal role in the proper formation of endothelial cell-cell contacts and barrier function (Fig. 6e). Our data underscore the importance of post-transcriptional regulation in endothelial homeostasis and might have implications for future therapeutic strategies aimed to preserve vascular integrity in health and disease.

## Methods

### Mice

C57BL/6 J mice were obtained from the Jackson Laboratory. Inducible Tg(Cdh5-cre/ERT2)1Rha (Jackson Laboratory) mice were crossed to B6.Cg-Gt(ROSA)26Sortm14(CAG-tdTomato)Hze/J (Jackson Laboratory) to obtain VE-cadherin-CreER/Rosa^tdTomato^ reporter mice. Tamoxifen (Sigma-Aldrich) was dissolved in a sunflower oil/ethanol (10:1) mixture at 10 mg/ml. Tamoxifen 2 mg was injected daily intraperitoneal into 8-week-old VE-cadherin-CreER/Rosa^tdTomato^ mice for 5 consecutive days. Quaking^viable^ (Qk^v^) mice were generously provided by dr. S. Richard, McGill University, Montreal, Canada. Quaking^viable^ mice have an autosomal recessive 1 Mb deletion in the promoter region of the Qki gene^59^,^60^. This results in decreased expression of QKI-6 and QKI-7 isoforms and serves as a well-recognized model to study QKI dependent effects *in vivo*. All animal use and experimental procedures were approved by the regulatory authorities of the Leiden University Medical Center and within the guidelines set by the Dutch government. All experiments were performed in accordance with relevant guidelines and regulations.

### Cell culture

HUVECs were isolated from human umbilical cords. The umbilical cords were collected and kept at 4 °C in PBS for a maximum of 3 days. To harvest the ECs, the vein was cannulated and flushed with sterile PBS. Thereafter, pre-heated (37 °C) trypsin-EDTA (Gibco) was incubated for 15 minutes in 37 °C PBS after which the vein was flushed with PBS again. The resulting cell suspension was spun down (1500 rpm, RT, 10 minutes), resuspended in Endothelial growth medium-2 (Lonza), and plated on 1% gelatin coated T75 flasks, cultured at 37 °C, 5% CO_2_. Experiments were performed with cell passage 1–3.

MVEC were a kind gift from Prof. M.J.T.H. Goumans, Leiden University Medical Center, Leiden, the Netherlands. MVECs are human pulmonary microvascular endothelial cells and were culture in microvascular endothelial cell medium-2 (EGM-2-MV, Lonza).

Human embryonic kidney cells (HEK293T) were cultured in DMEM (Gibco) supplemented with 100 U/ml penicillin, 100 U/ml streptomycin, 300 μg/ml glutamin (all from Gibco) and 10% fetal calf serum (Cambrex).

### Primary antibodies

Antibodies used to detect QKI-5, QKI-6 or QKI-7 were obtained from Millipore (rabbit polyclonals) or Neuromab (mouse monoclonals). MECA-32, VE-cadherin and NOS3 from BD Biosciences, GAPDH and β-catenin from Cell Signaling and β-actin from Abcam.

### Immunofluorescent staining

Cells cultured on glass coverslips or Ibidi 8-well μ-slides, cross-sections of aorta from VE-cadherin-CreER/Rosa^tdTomato^ mice, or cross-sections of kidney from C57BL/6 J mice were washed twice with Hanks’ Balanced Salt Solution containing Ca^2+^ and Mg^2+^ (HBSS, Gibco), fixed with 3.7% formalin (Millipore) for 10 minutes and permeabilized with 0.5% Triton X-100 for 2 minutes (Sigma-Aldrich) in HBSS. Indicated antibodies were incubated in HBSS containing 1% bovine serum albumin and 1% fetal calf serum for 1 hour. Appropriate secondary antibodies labeled with Alexa fluorophores were obtained from Invitrogen and phalloidin-rhodamine was obtained from Sigma-Aldrich. Cells or sections were mounted with Prolong^®^ gold antifade mountant with DAPI (Life technologies) and imaging was performed on an inverted Leica SP5 confocal imaging system.

### Quantitative RT-PCR

RNA isolation was performed using TRIZOL reagent (Invitrogen) and isolated using Qiagens’ RNAeasy kit according to manufactures instructions. 500–2000 ng of total RNA was used for reverse transcription mediated cDNA synthesis using random primers (Invitrogen) according to the manufactures protocols. SYBR Select (Invitrogen) and a Biorad CFX384 were used for qRT-PCR analysis (primers, Table 1). The change in mRNA expression was calculated by the comparative change-in-cycle-method (ΔΔC_T_).

### Immunoblot analysis

Protein lysates were generated using radioimmunoprecipitation buffer supplemented with protease inhibitors cocktail (Complete, Roche), total protein content was determined using BCA Protein Assay Kit (Pierce), and 5–20 μg of total protein was resolved using Any-kD Mini-PROTEAN TGX Precast SDS page gels (Biorad). Gels were blotted onto nitrocellulose membranes using the Trans-Blot Turbo Transfer System (Biorad) and blocked with 5% non-fat milk powder in PBS with 0.01% of Tween (PBST). Membranes were incubated with primary antibodies overnight at 4 °C, thereafter appropriate HRP-labeled secondary antibodies were incubated for 1 hour at room temperature and thoroughly washed with PBST. Next, membranes were incubated with SuperSignal West Dura Chemiluminescent Substrate (Pierce) and exposed on BioMax XAR Film (Kodak) or Ultracruz autoradiography film (Santa Cruz).

### Laminar shear stress experiments

Laminar shear stress experiments were performed using an Ibidi flow system. HUVECs were seeded onto closed perfusion chambers (IbidiTreat 0.4 μ-Slide I or VI, Luer) at a concentration of 1.5 × 10^6^ cells per ml. Cells were allowed to adhere for 3 hours and the chamber was connected to a computer-controlled air pressure pump and a fluidic unit with a two-way switching valve. The pump setup allowed pumping of 16 ml cell culture medium from two reservoirs in a unidirectional way through the flow channel over the monolayer of ECs at a constant shear stress of 10 dyne/cm^2^. The chamber and reservoirs containing the medium were kept in an incubator at 37 °C and 5% CO_2_. Medium was refreshed after 1 and 4 days of culture. RNA was isolated from cells not subjected to flow or subjected to shear stress for 7 days in a 0.4 μ-Slide I Luer flow chamber, while the 6 lanes of a 0.4 μ-Slide VI Luer were used for immunofluorescent stainings.

### Lentiviral vectors, lentiviral particle production and transduction

Four different shRNA constructs targeting human QKI were obtained from the Mission Library (Sigma Aldrich) and tested for their efficiency to knock down QKI. The best shRNA was selected to perform all experiments. As a control, a non-targeting shRNA was used. The human KLF2 overexpression construct was kindly provided by Prof. A. Horrevoets, VU University Medical Center, Amsterdam, the Netherlands. Lentiviral particles were produced as described by the Sigma Library protocol using HEK293T cells. Lentiviral transductions of HUVECs were done with cell passage 1 or 2. Lentiviral particles were incubated overnight and puromycin selection (0.5 μg/ml) for 24 hours was started 24 hours after lentiviral transduction.

### Endothelial barrier measurements

Endothelial barrier was measured by Electric-cell impedance sensing. HUVEC cultures were grown to confluency, where after the cells were detached using trypsin-EDTA solution, counted using trypan blue and seeded in the electrode arrays (Ibidi, 8 wells, 10 electrodes per well) coated with gelatin (1% in 0.9% NaCl) at a density of 50.000 cells/well. Measurements of trans-endothelial electrical resistance were performed in real time by means of an ECIS-Zθ instrument (Applied Biophysics) at 37 °C, 5% CO_2_. Cell spreading and monolayer formation were subsequently monitored by measuring the resistance at 4000 Hz. For the mathematical modelling to calculate resistance attributable to the functions cell-cell adhesion and cell-matrix interaction, the resistance and capacitance were measured at 11 different AC frequencies ranging from 62.5 Hz up to 64000 Hz.

### RNA immunoprecipitation

RNA-immunoprecipitation was performed using Millipore’s validated RIPAb + QKI-5 kit according to manufacture instructions.

### Luciferase assays

HEK293T cells were transfected with a human QKI promoter-luciferase reporter construct (SwitchGear Genomics), human β-catenin 3′UTR luciferase-reporter plasmid (SwitchGear Genomics), human VE-cadherin 3′UTR luciferase-reporter plasmid (last 545 bp of 3′UTR of human VE-cadherin cloned into pMIR-REPORT™ miRNA Expression Reporter Vector System; Applied Biosystems), control 3′UTR luciferase-reporter plasmid (60 bp of murine SDF-1 3′UTR cloned into pMIR-REPORT™ miRNA Expression Reporter Vector System; Applied Biosystems) or empty 3′UTR luciferase-reporter construct (SwitchGear Genomics) through the use of polyethylenimine (3 μg/1 μg DNA; Polysciences Inc). When indicated shRNA against QKI or non-targeting shRNA were co-transfected. Twenty-four hours after transfection luciferase activity was assessed with the Dual Luciferase Reporter reagents (Promega) and a GloMax^®^ 96 microplate luminometer (Promega) and was normalized to a constitutively expressed renilla reporter.

### *In vivo* vascular leakage

Female Quaking viable (Qk^v^) mice or wild type littermates aged 16–20 weeks old were anesthetized by intraperitoneal injection with 10 ml/kg of fentanyl (50 μg/ml), dexmedetomidine (Dexdomitor, 0.5 mg/ml), and midazolam (Dormicum, 5 mg/ml). Next, the mice were injected intravenous with 100 μl of 1% Evans Blue (Sigma-Aldrich) in PBS. After 15 minutes 8 mg/kg bradykinin was injected intravenous and mice were exsanguinated by cardiac puncture after 5 minutes. Plasma was collected, mice were perfused with 20 ml of PBS. The gut was taken out from stomach until the rectum, weighed, cut into small pieces and incubated overnight at 55 °C in 2 ml of Formamide (Sigma-Aldrich). Next, tubes were spun down, and supernatants were collected to determine light absorbance at 450 nm (reference) and 620 nm (Evans blue) wavelengths using a 96-wells plate in a spectrophotometer (SpectraMax, Molecular Devices). The Evans Blue OD/reference OD ratio per gram of wet tissue is given.

### Statistical analysis

The difference between two groups was analyzed by Student’s *t*-test. *P* values of less than 0.05 were considered significant.

## Additional Information

**How to cite this article**: de Bruin, Ruben G. *et al*. The RNA-binding protein quaking maintains endothelial barrier function and affects VE-cadherin and β-catenin protein expression. *Sci. Rep*. **6**, 21643; doi: 10.1038/srep21643 (2016).

## Supplementary Material

Supplementary Information

## Figures and Tables

**Table 1:** PCR Primer sequences.

	Forward (5′–3′)	Reverse (5′–3′)
Qki5	GACTGGCATTTCAATCCAC	GATGGACACGCATATCGTG
Qki6	GACTGGCATTTCAATCCAC	CGTTGGGAAAGCCATAC
Qki7	GACTGGCATTTCAATCCAC	GACTGGCATTTCAATCCAC
Klf2	CTACACCAAGAGTTCGCATCTG	CCGTGTGCTTTCGGTAGTG
Nos3	TGATGGCGAAGCGAGTGAAG	ACTCATCCATACACAGGACCC
VE-cadherin	GCACCAGTTTGGCCAATATA	GGGTTTTTGCATAATAAGCAGG
β-catenin	AGCTTCCAGACACGCTATCAT	CGGTACAACGAGCTGTTTCTAC
Gapdh	TTCCAGGAGCGAGATCCCT	CACCCATGACGAACATGGG
