# Supplementary material for: The RNA-binding protein quaking maintains endothelial barrier function and affects VE-cadherin and β-catenin protein expression
Source: Sci Rep. 2016 Feb 24;6:21643. doi: 10.1038/srep21643 (PMC4764852; doi:10.1038/srep21643)
Supplement: Supplementary Information [file srep21643-s1.pdf]

The RNA-binding protein quaking maintains endothelial barrier function and affects VE-cadherin and β-catenin protein expression

Ruben G. de Bruin, Eric P. van der Veer, Jurriën Prins, DaeHyun Lee, Martijn J. C. Dane, Huayu Zhang, Marko K. Roeten, Roel Bijkerk, Hetty C. de Boer, Ton J. Rabelink, Anton Jan van Zonneveld and Janine M. van Gils

Supplementary figures

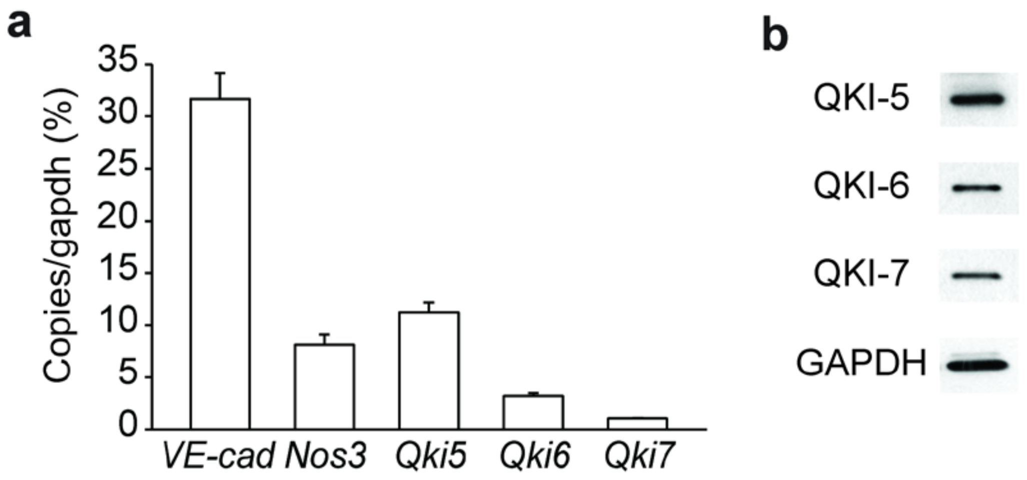

Supplementary figure 1. Quaking is highly expressed in HUVECs. (**a**) Quantitative RT-PCR analysis of VE-cadherin, Nos3, Qki5, Qki6 and Qki7 mRNA isolated from HUVECs. Results are presented as percentage of GAPDH. Mean ± s.e.m. of *n*=15. (**b**) Immunoblot analysis of QKI-5, QKI-6, QKI-7 or GAPDH (loading control) in protein lysates of HUVECs.

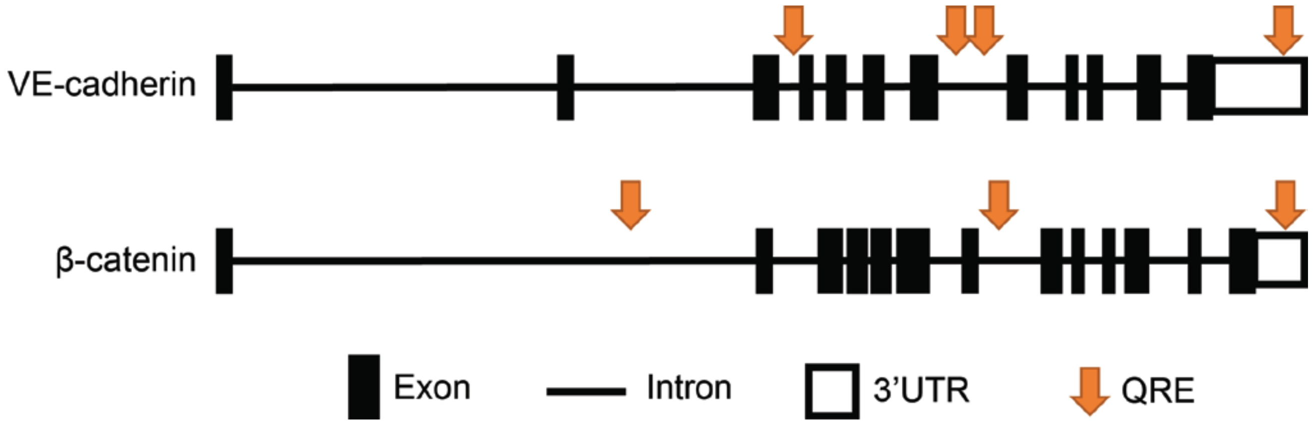

Supplementary figure 2. Quaking Response elements in VE-cadherin and ß-catenin. A schematic diagram of VE-cadherin and ß-catenin pre-mRNA with QKI response elements (QREs).

.

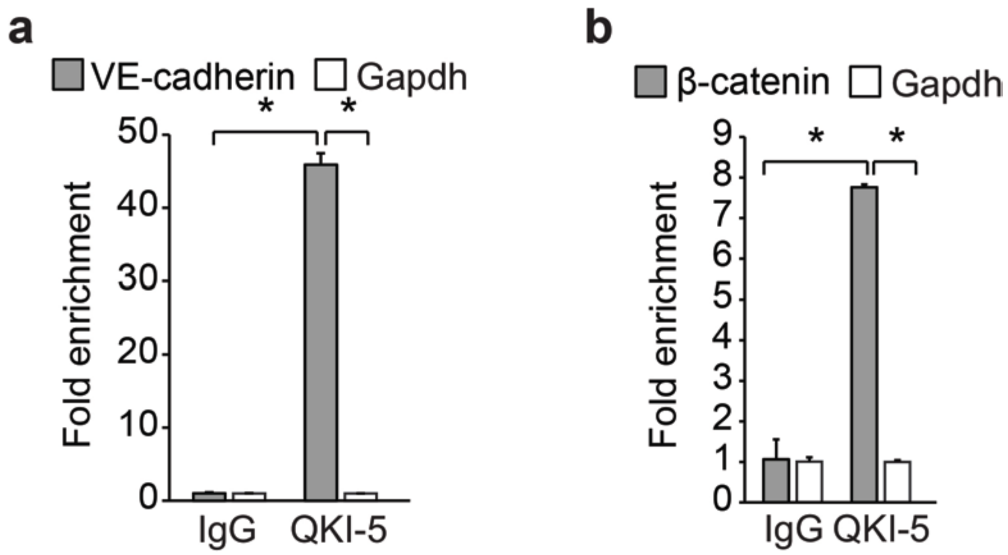

Supplementary figure 3. Quaking protein binds to VE-cadherin and β-catenin mRNA. (**a**-**b**) RNA-immunoprecipitation in MVECs using an IgG control or QKI-5 antibody. VE-cadherin (**a**), β-catenin (**b**) or Gapdh mRNA abundance in immune-precipitated fraction was determined by qRT-PCR. Results are presented relative to IgG immunoprecipitation, set as 1. Mean ± s.d. from one experiment representative of four independent experiments. *$P<0.05$.
